# Development of Thermoresponsive Hydrogels with Mucoadhesion Properties Loaded with Metronidazole Gel-Flakes for Improved Bacterial Vaginosis Treatment

**DOI:** 10.3390/pharmaceutics15051529

**Published:** 2023-05-18

**Authors:** Andi Dian Permana, Rangga Meidianto Asri, Muhammad Nur Amir, Achmad Himawan, Andi Arjuna, Nana Juniarti, Rifka Nurul Utami, Sandra Aulia Mardikasari

**Affiliations:** 1 Department of Pharmaceutical Science and Technology, Faculty of Pharmacy, Hasanuddin University, Makassar 90245, Indonesia; rangga.masri@farmasi.unhas.ac.id (R.M.A.); himawan@unhas.ac.id (A.H.); andiarjuna6854@gmail.com (A.A.); nanajuniartiunhas@gmail.com (N.J.); rifkanurulutami@unhas.ac.id (R.N.U.); sandramardikasari@gmail.com (S.A.M.); 2 Department of Pharmacy, Faculty of Pharmacy, Hasanuddin University, Makassar 90245, Indonesia; nuramir@unhas.ac.id

**Keywords:** metronidazole, bacterial vaginosis, gel flakes, thermoresponsive hydrogels, mucoadhesive

## Abstract

Bacterial vaginosis is an infectious disease that has significantly affected women’s health. Metronidazole has been widely used as a drug for treating bacterial vaginosis. Nevertheless, the currently available therapies have been found to be inefficient and inconvenient. Here, we developed the combination approach of gel flake and thermoresponsive hydrogel systems. The gel flakes were prepared using gellan gum and chitosan, showing that the incorporation of metronidazole was able to provide a sustained release pattern for 24 h with an entrapment efficiency of >90%. Moreover, the gel flakes were incorporated into Pluronics-based thermoresponsive hydrogel using the combination of Pluronic F127 and F68. The hydrogels were found to exhibit the desired thermoresponsive properties, showing sol-gel transition at vaginal temperature. Following the addition of sodium alginate as a mucoadhesive agent, the hydrogel was retained in the vaginal tissue for more than 8 h, with more than 5 mg of metronidazole retained in the ex vivo evaluation. Finally, using the bacterial vaginosis infection model in rats, this approach could decrease the viability of *Escherichia coli* and *Staphylococcus aureus* with reduction percentages of more than 95% after 3 days of treatment, with the healing ability similar to normal vaginal tissue. In conclusion, this study offers an effective approach for the treatment of bacterial vaginosis.

## 1. Introduction

Bacterial vaginosis (BV) is the disruption of the vaginal microbiome in which a variety of BV-associated species replace the generally dominating species of *Lactobacillus*. Increased vaginal discharge and unpleasant smell are the most common characteristics of this infection. The vaginal pH is elevated to more than 4.5 during BV [1,2]. In this disease, natural disinfection is provided by the lactobacilli in the vagina that form hydrogen peroxide. The lactobacilli-produced lactic acid aids in preserving the vagina’s natural pH and microbial balance [3]. Lactobacilli also create lactic acid, hydrogen peroxide, and bacitracin (an antibiotic peptide). The most common cause of BV is *Escherichia coli*, a Gram-negative anaerobic bacteria often present in the lower intestine. BV can occur due to the migration of this bacteria to the vagina. Under normal conditions, the vaginal mucosal membrane often offers a defense against this bacterial migration [2,4]; however, the mucosal lining may deteriorate as a result of the protection being undermined by an insufficient diet, low hormone levels, poor health, frequent sexual activity, or aberrant microbiota. It is conceivable that women with BV who often engage in sexual activity might have urinary tract infections. Urinary tract infections are mostly brought on by *Escherichia coli* [2]. In addition, it has been also reported that *Staphylococcus aureus* is another microorganism causing BV [5,6].

Metronidazole, an antibiotic with high activity towards anaerobic bacteria, is one of the most preferred treatments for BV [7,8]. Both oral and intravaginal dosage forms of metronidazole are available for BV treatment [1,2]. Vaginal cream and pessaries are among the examples of the intravaginal formulations. Conventionally, cream is the popular choice for topical administration on account of its simplicity and excellent spreadability. Yet, cream may cause inconvenience to be used intravaginally due to its greasiness [9,10]. Meanwhile, utilizing vaginal pessaries results in many problems, such as patient discomfort and allergy concerns. In the past, a number of studies have been carried out to create a locally targeting metronidazole [11,12,13,14]. A gel-based formulation is superior to cream as an alternative for topical administration because it has non-sticky properties and has a straightforward manufacturing process [15]. Unfortunately, the high water content of conventional gel caused it to be easily rinsed away by vaginal physiological fluid during application. In situ thermoresponsive gel is created employing thermogelling polymers, which enable the conversion of a free-flowing fluid into a solid gel at physiological temperature, to solve this issue [16,17,18,19,20]. As a result, this study proposes in situ vaginal gel to give prolonged contact with the mucosal lining of vagina, increasing medication absorption and therapeutic effectiveness. The effectiveness of this particular system has been widely reported in numerous studies previously [18,21,22,23,24,25].

In this work, metronidazole was added to the gel flakes system before the thermoresponsive hydrogel fabrication. Gel flakes are polygonal fibroid structures that can increase the likelihood of metronidazole adhering to the densely folded epithelial surfaces of the vagina, resulting in extended interaction with vaginal mucus and controllable drug release [26,27]. The gel flake system was then incorporated with thermoresponsive polymers to further improve the local targeting efficacy of metronidazole, which is presented for the first time in this report. In this study, to facilitate the administration of the gel flakes, we incorporated the formulation into a thermoresponsive hydrogel with mucoadhesive properties. The mucoadhesive properties are crucial in vaginal application since the presence of vaginal fluid can rinse the formulation applied intravaginally. Therefore, the use of a mucoadhesive agent could be beneficial to improve the formulation residence time in the vagina, increasing the treatment efficacy. Specifically, to develop the thermoresponsive hydrogel, we used Pluronics. This polymer is synthetic triblock copolymers with poly(ethylene oxide)-b–poly(propylene oxide)-b–poly(ethylene oxide) (PEO–PPO–PEO) chains. The combination two Pluronics, namely PF-127 and PF-68, were chosen due to the ability of these polymers to exhibit sol-gel transition at a specific temperature [28]. Several studies have shown that the combination of these two polymers could result in T_sol-gel_ at body temperature with better properties compared to a single polymer [29,30,31,32]. As a mucoadhesion polymer, a natural carbohydrate derivatives polymer, sodium alginate, was added into the thermoresponsive hydrogels. In addition, a number of comprehensive evaluations were conducted following gel formulations, including physicochemical characterization, an ex vivo penetration and retention test using vaginal tissue, and in vivo experiments using suitable animal models.

## 2. Materials and Methods

### 2.1. Materials

Metronidazole, gellan gum, chitosan, and Pluronic^®^ F127 (PF127) were purchased from Sigma-Aldrich Pte Ltd. (Singapore). Pluronic^®^ F68 (PF68) was generously provided by BASF SE (Jakarta, Indonesia). All other compounds in this study were pharmaceutical grade.

### 2.2. Formulation of Metronidazole-Loaded Gel Flakes

The formulation of gel flakes containing metronidazole was performed using several compounds, as shown in Table 1. Initially, gellan gum was dissolved in boiled water. After being completely dissolved, the temperature was cooled to room temperature. After that, metronidazole was mixed with gellan gum solution. The mixture was added dropwise to chitosan solution (in 1% acetic acid) while being stirred at 200 rpm at 25 °C.

### 2.3. Characterization of Metronidazole-Loaded Gel Flakes

The encapsulation efficiency (EE) of metronidazole in gel flakes matrices was determined using indirect method. The formulation was filtered using a 0.2 μm syringe filter. Afterwards, the free metronidazole in the filtrate was analyzed using UV–vis spectrophotometer at 320 nm. Finally, the EE values was calculated in three replicates using the equation below [27]

(1) $$\%EE=\frac{W_{1}-W_{2}}{W_{1}}\times 100$$

where $W_{1}$ is the concentration of metronidazole in the formulation and $W_{2}$ is the concentration of metronidazole detected in the filtrate.

The determination of drug loading (DL) capacity of metronidazole-loaded with gel flakes started with dispersing 25 mg of the formulation in 50 mL of distilled water. After that, the mixture was sonicated for 20 min. The mixture was then centrifuged for 20 min at 7000 rpm. The concentration of metronidazole was measured using UV–vis spectrophotometer at 320 nm. Finally, the DL was calculated in three replicates using the equation [33]

(2) $$\%DL=\frac{Amount\;of\;metronidazole\;detected}{Total\;weight\;of\;the\;formulation}\times 100$$

The possible interaction between metronidazole and the excipients used in this study was investigated using Fourier transform infrared (FTIR) spectrometer (Shimadzu^®^ FTIR-8400, Shimadzu, Kyoto, Japan). The analysis was performed at room temperature with a resolution of 4.0 cm^−1^ between 400 and 4000 cm^−1^ using 32 scans.

Different scanning calorimetry (DSC) Q100 (DSC 2920, TA Instruments, Surrey, UK) was utilized to evaluate the thermal properties of metronidazole in the gel flakes formulation. The samples were sealed in an aluminum pan and analyzed with a heating rate of 10 °C/min from 0 to 300 °C.

Briefly, the gel flakes were dried at 37 °C. The dry gel flakes were placed into carbon adhesive tape. Finally, a scanning electron microscope (SEM) (JEM-1400Plus; JEOL, Tokyo, Japan) was used to observe the morphologies of gel flakes containing metronidazole.

### 2.4. In Vitro Release Studies of Metronidazole from a Gel Flakes Formulation

In this study, a dialysis technique was used. In this study, in an attempt to mimic the vaginal environment, the release study of metronidazole from gel flakes was performed in the simulated vaginal fluid. The fluid was prepared using NaCl (3.51% *w*/*v*), KOH (1.4% *w*/*v*), Ca(OH)_2_ (0.22% *w*/*v*), bovine serum albumin (0.018% *w*/*v*), lactic acid (2% *w*/*v*), acetic acid (1% *w*/*v*), glycerol (0.16% *w*/*v*), urea (0.4% *w*/*v*), and glucose (5% *w*/*v*). The pH of the solution was adjusted to 4.6. Metronidazole and metronidazole-loaded gel flakes (equivalent to 50 mg of metronidazole) were placed inside the dialysis membrane (Spectra-Por^®^, 12,000–14,000 MWCO) (Spectrum Medical Industries, Los Angeles, CA, USA). The membrane was put in 100 mL of simulated vaginal fluid in an orbital shaker. The study was carried out at 37 °C at 100 rpm. At interval times (0.5 h, 1 h, 2 h, 3 h, 4 h, 5 h, 6 h, 7 h, 8 h, 12 h, and 24 h), 1 mL of sample was collected, and the concentration of metronidazole was determined using spectorphotometry. To ensure that sink condition was achieved, the media were replaced with fresh fluids after each collection. Following this, different release kinetic models, namely zero-order, first-order, Higuchi, Korsmeyer–Peppas, and Hixson Crowell [34] were applied to the release profiles. DDSolver was used to analyze the kinetic models. The experiment was conducted in three replicates.

### 2.5. Preparation of Mucoadhesive-Thermoresponsive In Situ Hydrogel Containing Metronidazole Gel Flakes

The gel flakes of metronidazole were incorporated into mucoadhesive and thermoresponsive hydrogel formulation. The formulations were developed using PF-127 and PF-68 as thermogelling compounds and sodium alginate was used as a mucoadhesion compound. The formulation components are depicted in Table 2. The cold method was used to prepare the hydrogel [25]. Initially, the thermogelling agents were dissolved in distilled water at 5 °C. In a separate container, sodium alginate was dissolved in distilled water and mixed with a thermogelling solution. Following this, the gel flakes were mixed with the hydrogel for 30 min under stirring (200 rpm).

### 2.6. Characterization of Mucoadhesive-Thermoresponsive In Situ Hydrogel Containing Metronidazole Gel Flakes

#### 2.6.1. The Determination of Gelation Temperature (T_sol-gel_)

The gelation temperature (T_sol-gel_) was determined by tube inversion method [35]. Initially, 2 mL of hydrogel was placed in a glass tube at 4 °C. The tube was immersed in water at 20 °C and the temperature was gradually increased by 1° each time. The gelation temperature was denoted when the gel successfully formed and did not flow freely when the tube was tuned over 90° for 30 s. Additionally, the T_sol-gel_ of the formulation after being diluted with vaginal fluid was also evaluated in three replicates.

#### 2.6.2. Mucoadhesion Strength

A modified physical balance was applied to determine the mucoadhesion ability of the formulation, with slight modification [36]. In this study, a fresh porcine vaginal tissue was used and attached between two glass vials. To set the experiment, the first vial was attached to the balance and the second vial was put on a height-adjustable pan of the balance. The hydrogel was applied in the mucosal tissue and the vials were connected tightly for 2 min. Finally, the metal weights were added into the other pan of the balance. The weight required to detach the vials was noted and the strength of the mucoadhesion ability was then determined in three replicates using the equation

(3) $$Mucoadhesive\;strength\;\left(\mathrm{dyne}\cdot {\mathrm{cm}}^{2}\right)=\frac{m\cdot g}{A}$$

where *m* is the minimum weight required to detach the vials (g), *A* is the average area of vaginal mucosal (cm^2^), and *g* is the gravity force (980 cm/s^2^).

#### 2.6.3. Mucoadhesion Time

In this study, the type 2 dissolution USP apparatus was used to evaluate the mucoadhesion time of the hydrogel [37]. A fresh porcine vaginal tissue was attached on the paddle of the dissolution apparatus. Afterwards, 1 g of hydrogel formulation was applied to the vaginal mucosal. Then, 900 mL of simulated vaginal tissue was placed inside the dissolution jar. The study was performed in three replicates at 100 rpm at 37 °C. The time required by the hydrogel to detach from the vaginal tissue was recorded as the mucoadhesion time.

#### 2.6.4. Investigation of Viscosity and Rheological Properties

The viscosity and the rheology properties of the hydrogel were evaluated using DV-III Ultra viscometer (RV model, Brookfield, WI, USA). Specifically, three different temperature conditions were used to measure the viscosity of the hydrogel, namely storage temperature (4 °C), room temperature (25 °C), and physiological temperature (37 °C). The experiment was performed in three replicates using spindle 07 at 500 rpm.

#### 2.6.5. Determination of pH and Drug Content Analysis

The pH of the hydrogel formulation was measured in three replicates using a digital pH meter (Horiba Scientific, Kyoto, Japan). The determination was carried out at 25 °C.

The recovery of metronidazole in the hydrogel formulation was investigated by dissolving 100 mg of the hydrogel in 100 mL of methanol under sonication condition for 1 h. Then, the samples were centrifuged for 15 min at 7000 rpm. The concentration of metronidazole was analyzed using spectrophotometry in three replicates [33].

### 2.7. Ex Vivo Permeation Studies

This study was approved by the Ethical Committee from the Faculty of Medicine, Hasanuddin University, Makassar, Indonesia (Number UH20070336). The ex vivo permeation ability of metronidazole through vaginal tissue was assessed using a Franz diffusion cell with an area of 4.9 cm^2^. The fresh porcine vaginal mucosa was attached between the donor and the receptor compartments. Simulated vaginal fluid (12 mL) was used as the medium in the receptor compartment. Afterwards, 1 mL of the hydrogel was applied to the donor compartment. This experiment was carried out at 37 ± 1 °C at 100 rpm. At a predetermined time (0.5 h, 1 h, 2 h, 3 h, 4 h, 5 h, 6 h, 7 h, and 8 h), 1 mL of the medium was taken and replaced immediately with fresh media [37]. The concentration of metronidazole was measured using spectrophotometry in three replicates.

### 2.8. Ex Vivo Retention Determination

At the end of the permeation study, the vaginal tissue was collected and any excess of the formulation was removed from the surface using distilled water. Metronidazole was extracted from the tissue using 10 mL methanol under sonication for 30 min. After that, the mixture was centrifuged at 7000× *g* rpm for 15 min. The supernatant was collected and the concentration of metronidazole was determined using a UV–vis spectrophotometer in three replicates [37]. It was important to note that the analytical method used in this study was validated and found to be selective, precise, and accurate with the presence of other substances, including the vaginal tissue.

### 2.9. In Vivo Antibacterial Activity in Model of Infection on Rat

#### 2.9.1. Preparation of Bacterial Vaginosis Model on Rat

The ethical clearance for this study was granted by the Ethical Committee from the Faculty of Medicine, Hasanuddin University, Makassar, Indonesia (Number UH20070336). Female Wistar rats were used in this study and underwent acclimatization for 7 days in the laboratory environment. To develop the infection model, 20 µL of *Escherichia coli* and *Staphylococcus aureus* with bacterial number of 1 × 10^6^ CFU/mL was applied daily to the rat’s vaginal cavity for 3 days [38].

#### 2.9.2. In Vivo Antibacterial Activity and Histopathology Evaluation

In this study, the rats were divided into four cohorts, namely hydrogel containing gel flakes of metronidazole, hydrogel containing free metronidazole, hydrogel without drug, solution of metronidazole, dispersion of metronidazole gel flakes, and negative control. Initially, 1 g of the formulation was administered intravaginally to the animals twice daily. At predetermined time points, the vaginal fluid (1 μL) was collected and inoculated into Eosin Methylene Blue Agar and Vogel Johnson Agar to quantify *Escherichia coli* and *Staphylococcus aureus*, respectively. The media were incubated at 37 °C for 24 h and the bacterial numbers were calculated, expressed as CFU/mL [23]. The in vivo study was carried out for 3 days in three replicates. At the end of the experiment, the vaginal sample rats were excised for the evaluation of the histopathology. The assessment was conducted using the hematoxylin eosin staining.

### 2.10. Statistical Analysis

All data were presented as mean ± SD. All data were analyzed using GraphPad Prism 6.0 (GraphPad, San Diego, CA, USA). To obtain the conclusion, *p* < 0.05 was noted as a significant result.

## 3. Results and Discussion

### 3.1. Formulation and Characterization of Metronidazole Loaded Gel Flakes

In this study, to modulate the release of metronidazole, the drug was developed into a gel flakes-based approach. This system can be formed due to the gelation reaction between the anionic part of carboxylate ions contained in gellan gum and the cationic part of amino groups contained in chitosan [27]. This will subsequently entrap the drug between the polymer matrix, hence controlling the release. To characterize the formulation, initially, we assessed the EE percentage of metronidazole in the gel flakes formulation. It was found that the EE values ranged from 77.67 ± 3.43% to 99.47 ± 5.87%, as shown in Table 3. The results showed that the increase in gellan gum and chitosan concentrations could increase the EE values of the formulation. It was because the increment of these two polymers could increase the gelation capacity, resulting in the high entrapment of metronidazole in the formulation of gel flakes.

Furthermore, we assessed the DL of metronidazole in the gel flakes formulation, as shown in Table 3. We found that the difference between the EE and the formulation composition affected the DL of metronidazole. In this study, the optimized formulation was chosen by the high EE and DL values. Analyzed statistically, F5 and F6 showed the highest EE values and were significantly higher (*p* < 0.05) than those of other formulations. Meanwhile, with respect to DL value, since F6 contained higher amount of gellan gum, its DL value was significantly lower (*p* < 0.05) than F5. Accordingly, F6 was selected for further studies.

**Figure 1:**
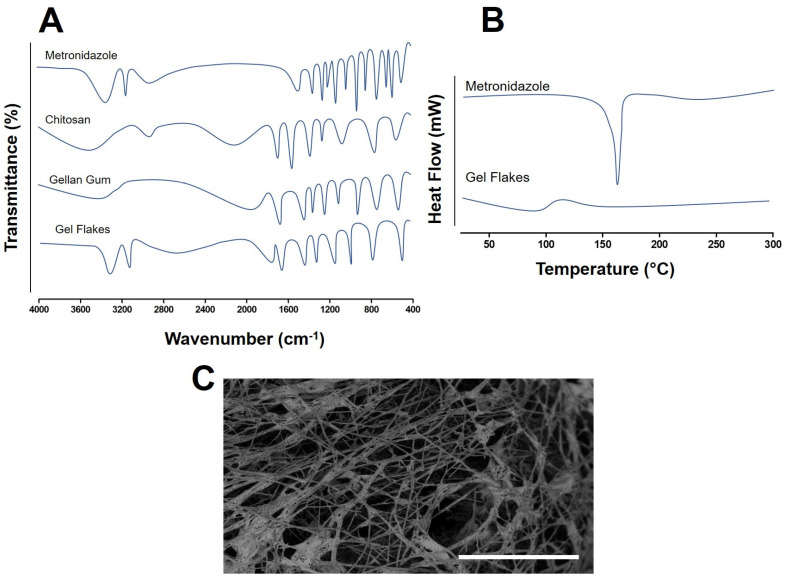
FTIR spectrum of metronidazole, chitosan, gellan gum, and gel flakes (**A**). DSC thermogram of metronidazole and gel flakes formulation (**B**). The SEM images of gel flakes formulation containing metronidazole (scale 50 μm) (**C**).

In FTIR evaluation (Figure 1), with respect to metronidazole spectrum, several peaks were observed. The stretching detected at 3221 cm^−1^ was due to the presence of OH group of the drug. The peaks at 3110 cm^−1^ and 1533 cm^−1^ were found, representing C=CH and NO_2_/N-O, respectively. At 1193 cm^−1^, the spectrum indicated the stretching vibration of the tertiary amine group. Finally, the presence of C-OH/C=O and C-NO_2_ were identified by the peaks at 1071 cm^−1^ and 884 cm^−1^, respectively. In the chitosan spectrum, the peaks were found at 1661 cm^−1^ and 1595 cm^−1^ due to the presence of C=O and N-H, respectively; however, these peaks were found to shift to 1635 cm^−1^ and 1575 cm^−1^. This could be due to the interaction between gellan gum and chitosan, resulting in the change of the environment of the amine group. A similar trend was also found in the previous study, showing the successfulness of the formation of the gel flakes [26,27]. For gellan gum, the presence of asymmetric carboxylate anion stretching resulted in peak detected at 1604 cm^−1^. Moreover, peaks at 1410 cm^−1^ presented the presence of symmetric carboxylate anion. These two peaks were shifted to 1641 cm^−1^ and 1445 cm^−1^, due to the gelation reaction. The changes in the FTIR spectrum indicated the successfulness of the formation of gel flakes. Moreover, it is evident that the peaks of metronidazole did not change in the gel flakes formulation, indicating that the formulation did not affect the structure of metronidazole.

The thermal property of metronidazole was then investigated using DSC. The thermograms of metronidazole and gel flakes formulation are depicted in Figure 1. In the metronidazole thermogram, the sharp peak was detected at 160 °C, indicating the melting point and the crystallinity of metronidazole. In the gel flakes formulation, the peak disappeared; this possibly demonstrated that metronidazole might change to an amorphous form. In this study, it could be hypothesized that the formation of the amorphous state was initially induced by mixing the drug with the polymers, removing the water which further formed the dry state. Following the mixing of the polymer and the drug, the chains of the polymer experience a larger state of disorder. This could finally circumvent the crystal growth of the drugs mixed with the polymers [39]. Additionally, this also showed that metronidazole was completely encapsulated inside the gel flakes matrix. This indicated that the method used to form the gel flakes effectively loaded the drugs into the formulation. Therefore, this could potentially enhance the controlled release of metronidazole from the formulation into the vaginal environment [26]. Regarding the morphology analysis using SEM, the result showed that the flakes were formed in polygonal structures and small size (Figure 1). This structure was preferred in the vaginal administration as this form could potentially penetrate and spread into the highly gathered vaginal surfaces.

### 3.2. In Vitro Release Studies of Metronidazole from Gel Flakes Formulation

**Figure 2:**
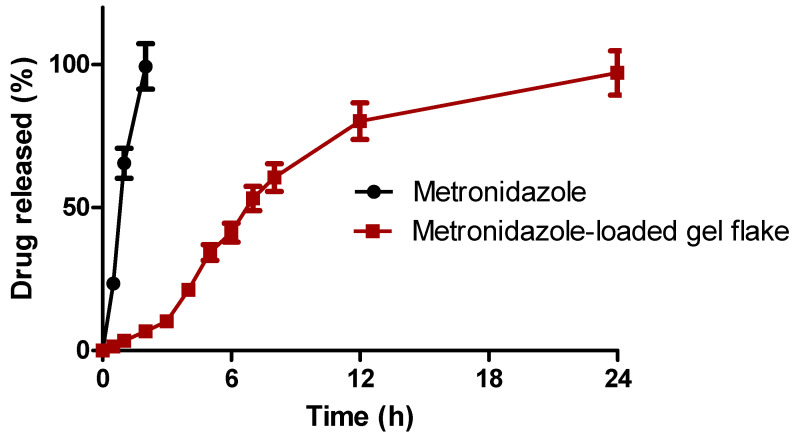
In vitro release of pure metronidazole and metronidazole-loaded gel flake (mean ± SD, *n* = 3).

Here, gel flakes were developed to control and sustain the release of metronidazole. Therefore, we further evaluated the in vitro release study of metronidazole from gel flakes. The release was compared to the pure metronidazole and the results of this study are depicted in Figure 2. As shown, it was found that after 2 h, 99.37 ± 4.76% of metronidazole was completely released in the simulated vaginal fluid media. On the other hand, following the incorporation into gel flakes formulation, the release of metronidazole was sustained over 24 h, showing the release percentage of 97.11 ± 3.87%. This showed that the gelation reaction between two polymers used in the preparation of gel flakes could act as a matrix to control the release of metronidazole. Following this, the release mechanism of metronidazole from gel flakes was assessed. After the calculation, the release mechanism followed the Higuchi model with an r value of 0.976. Hence, it could be concluded that the release of metronidazole from gel flakes matrix is based on the erosion and the degradation of the matrix of the formulation.

### 3.3. Formulation and Characterization of Mucoadhesive-Thermoresponsive In Situ Hydrogel Containing Metronidazole Gel Flakes

#### 3.3.1. Results of Gelation Temperature (T_sol-gel_) Determination

**Figure 3:**
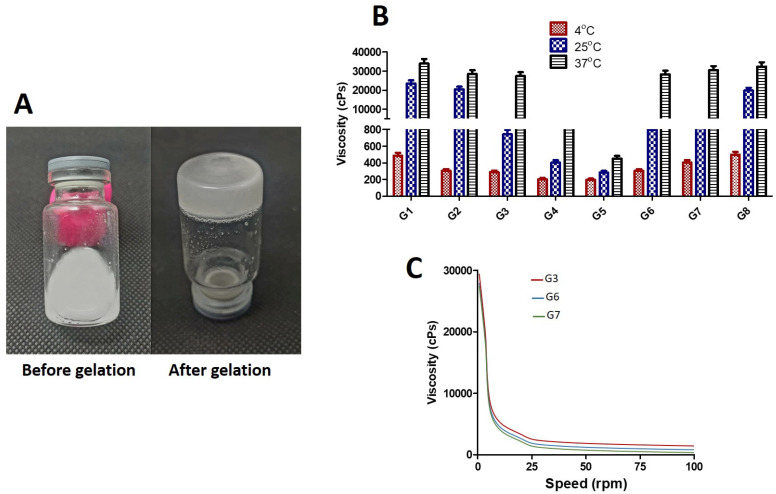
The representative images of hydrogel formulation before and after gel transition (**A**). The viscosity of thermoresponsive hydrogels at different temperatures (**B**) (mean ± S.D., *n* = 3) and the rheology pattern of thermoresponsive hydrogel (**C**).

This step was carried out to ensure that the hydrogel could be in the liquid form at the room temperature and could change to gel form when being applied at the vaginal temperature. Figure 3A exhibits the representative images of hydrogel formulation before and after gel transition. In the vaginal administration, it should be borne in mind that the composition of vaginal fluid itself may affect the gelation temperature of the hydrogel. Accordingly, in addition to the determination of the gelation of the hydrogel itself, it was crucial to evaluate the T_sol-gel_ of the formulation after being diluted with simulated vaginal fluid. It was found that compared to other formulations, formulation G3, containing the combination of PF-127 and PF-68 with the ratio of 15% and 5%, respectively, possessed T_sol-gel_ at the vaginal temperature, which was desired in this study. Importantly, after dilution with a simulated vaginal fluid, this formulation could maintain its T_sol-gel_ value. There was no statistically significant difference (*p* > 0.05) between the T_sol-gel_ values without and with dilution. The result demonstrated that the increase in PF-127 concentration could decrease the gelation temperature of the hydrogels, due to the longer triblock chain of PF-127 (Table 4). After the addition of sodium alginate as mucoadhesive agent, the T_sol-gel_ of the hydrogel was observed. The results showed that 0.2% and 0.4% of sodium alginate (G6 and G7, respectively), did not affect the T_sol-gel_ values significantly (*p* > 0.05). However, the use of 0.6% of sodium alginate (G8) decreased the T_sol-gel_ value of the hydrogel significantly (*p* < 0.05), which made this system unsuitable as thermoresponsive system. Therefore, 0.6% of sodium alginate was not suitable to be used in the development of the desired system.

#### 3.3.2. Results of Mucoadhesion Strength and Mucoadhesion Time Determination

To avoid the removal of the formulation after the administration into the vaginal cavity, we added sodium alginate in the thermoresponsive hydrogel. The results showed that without the use of sodium alginate, the mucoadhesive strength and time were found to be relatively low. As shown in Table 5, the addition of sodium alginate increased both mucoadhesive parameters significantly (*p* < 0.05). The mucoadhesion occurred due to the hydrogen bonding formation between glycoprotein of mucin in the mucosal tissue and the -COOH group of cellulose [40]. Furthermore, the increase in sodium alginate concentration could increase the mucoadhesivity properties (strength and time) of the hydrogels in the vaginal tissue. Specifically, in G6 and G7, we found that the use of 0.2% and 0.4% of sodium alginate increased the mucoadhesion parameters significantly (*p* < 0.05). Interestingly, increasing the concentration of sodium alginate further to 0.6% did not increase the mucoadhesion properties significantly (*p* > 0.05). As previously explained, this concentration also changed the T_sol-gel_ temperature of the hydrogel.

#### 3.3.3. Viscosity and Rheological Study

In this study, we further investigate the viscosity and rheological behavior of the resulting hydrogels. The viscosity measurements were in good agreement wih the T_sol-gel_ determination. Without the use of sodium alginate, only G3 could show a desired trend of the viscosity values at 4 °C, 25 °C, and 37 °C (Figure 3). The formulation possessed liquid viscosity at the cold and room temperatures, while showing high viscosity in the vaginal formulation. The addition of sodium alginate with concentration of 0.2% and 0.4% did not change the viscosity properties significantly (*p* > 0.05). With regard to the rheological behavior, the liquid being free-flowing at room temperature for the ease of administration while converting into gel at vaginal temperature was desired in this study. With respect to the flow behavior, because the approach showed pseudoplastic performance, consequently, the thermoresponsive hydrogel should form shear-thinning properties in both liquid and gel forms. As shown from the results, the viscosity reduced with the increasing shear rate. The high shear rate could breakdown the three-dimensional assemblies leading to the reduction in the viscosity [41]. As shown in Figure 3, all hydrogels exhibited this desired condition.

#### 3.3.4. pH Measurement and Drug Content Analysis

Another important factor for patient’s convenience for the use of vaginal dosage form is pH. Normally, the pH should be around the pH of the vagina, which are, typically, between 4.5 and 5.5. This is important to avoid the possibility of any irritation during the administration. The results of pH measurement of the hydrogel formulation are shown in Table 6. The results indicated that all hydrogels possessed pH values in the vaginal pH, ranging from 4.76 to 5.44. Accordingly, the administration of the hydrogel would not cause irritation to the vaginal tissue [20,42].

It was crucial to ensure that the formulation process did not affect the active compound concentration. The ideal value for drug recovery is 95–105% [43]. In this study, the recoveries of metronidazole in the hydrogel formulations were between 97.76 and 99.63%. It can be concluded that the formulations were homogenous and the production process did not affect the concentration of metronidazole in the final formulations.

### 3.4. Ex Vivo Permeation Studies

**Figure 4:**
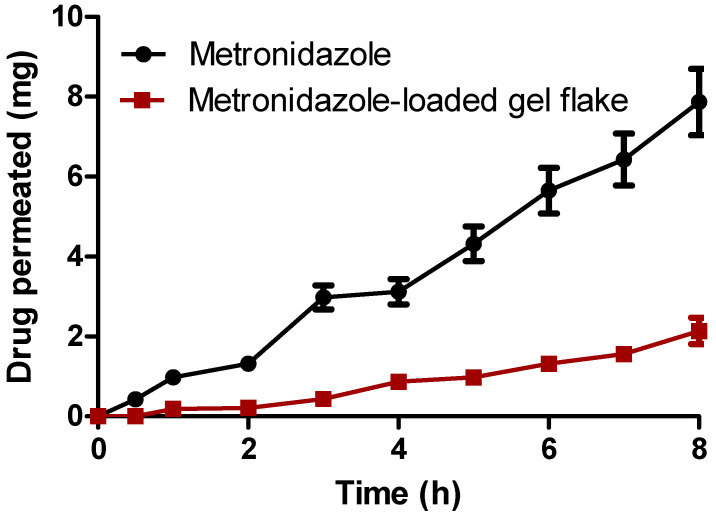
Ex vivo permeation profiles of metronidazole and metronidazole-loaded gel flake from thermoresponsive (mean ± S.D., *n* = 3).

The permeation ability of metronidazole from the thermoresponsive hydrogel through the vaginal tissue was then evaluated. In this study, based on the previous results of the characterization of the hydrogel, G7 was selected for the evaluation. As a comparison, the thermoresponsive hydrogel containing free metronidazole was also evaluated for the ex vivo permeation study. Figure 4 showed the amount of metronidazole penetrating the vaginal tissue from the hydrogel formulations containing gel flakes of metronidazole and free metronidazole. It was clearly seen that without the incorporation into the gel flakes system, the amount of metronidazole detected in the receptor compartment was found to be relatively high, achieving 7.87 ± 0.83 mg after 8 h. On the other hand, when metronidazole was incorporated into gel flakes, after 8 h, the concentration of metronidazole was significantly lower (*p* < 0.05) with the release amount of 2.14 ± 0.33 mg. In the bacterial vaginosis, the drug should not achieve the systemic circulation as the main objective of the treatment is to localize the drug in the vaginal tissue. Therefore, based on the results obtained in this study, the incorporation of the formulation of gel flakes could potentially decrease the amount of metronidazole reaching the systemic circulation.

### 3.5. Ex Vivo Retention Determination

The main aim of this study was to localize metronidazole in the vaginal tissue for the treatment of BV. Instead of the amount of metronidazole permeating the vaginal tissue, the amount of metronidazole retained in the vagina was more critical. After the permeation study, the concentration of metronidazole retained in the tissue was also quantified. The concentration of metronidazole in the vaginal tissue after 8 h was found to be 5.67 ± 0.66 mg following the administration of hydrogel containing gel flakes. Meanwhile, only 0.67 ± 0.08 mg of metronidazole was retained after the administration of hydrogel containing free drug. Accordingly, the incorporation of metronidazole in the gel flakes system and delivered using thermoresponsive-mucoadhesive hydrogel could not only avoid the systemic exposure oof metronidazole, but also improve the drug accumulation in the vaginal tissue, resulting in the improvement of bacterial vaginosis.

### 3.6. In Vivo Antibacterial Activity and Histopathology Evaluation in Model of Infection on Rat

**Figure 5:**
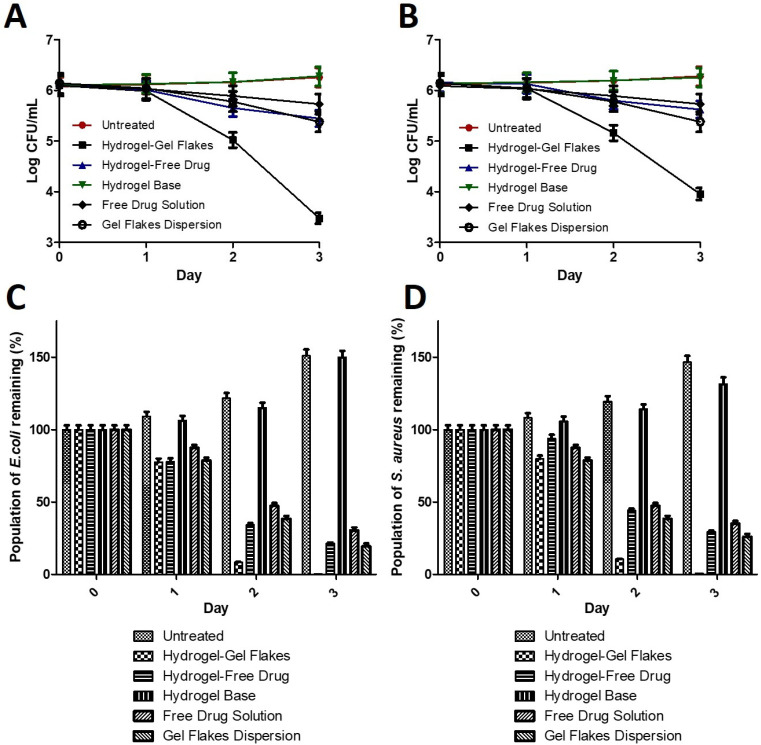
The viability of *E. coli* (**A**) and *S. aureus* (**B**) following several treatments (mean ± S.D., *n* = 3). The population of *E. coli* (**C**) and *S. aureus* (**D**) remaining in the vaginal tissue following several treatments (mean ± S.D., *n* = 3).

Finally, to prove the efficacy of the system developed in this study, we performed in vivo antibacterial activity in a bacterial vaginosis model. As a bacterial model, *Escherichia coli* and *Staphylococcus aureus* were used to create bacterial vaginosis model in female rats. In this study, the efficacy of our approach was compared to several groups, namely the hydrogel containing free metronidazole, the hydrogel base, and the untreated group. As shown in Figure 5, following the administration of the hydrogel containing gel flakes of metronidazole, after 3 days of treatment, the bacterial number dropped from 6.09 Log CFU/mL to 3.48 log CFU mL and from 6.13 Log CFU/mL to 3.95 Log CFU/mL for *Escherichia coli* and *Staphylococcus aureus*, respectively. This indicated the bacterial burden reduction of 99.76% for *Escherichia coli* and 99.35% for *Staphylococcus aureus*. On the other hand, without being formulated into gel flakes, although the hydrogel containing free metronidazole still showed antibacterial activity, this approach could only decrease the bacterial burden to 78.63% for *Escherichia coli* and 70.63% for *Staphylococcus aureus*. We also compared the antibacterial activity of our approach with free drug solution and gel flakes dispersion. The administration of both systems could reduce the bioburden of *Escherichia coli* up to 69.55% and 79.76%, respectively. In *Staphylococcus aureus* infection models, the administration of free drug solution and gel flakes dispersion was able to reduce the bacterial bioburden up to 65.78% and 74.28%, respectively. Therefore, this shows the significance of gel flakes formulation. In a good agreement with ex vivo study, due to the ability of gel flakes to localize metronidazole in the vaginal tissue, in an in vivo study, it was confirmed that this system could show significantly higher (*p* < 0.05) antibacterial activity in the bacterial vaginosis model. In contrast, the untreated group and hydrogel base group did not show any decrease in the bacterial number after 3 days, showing that we successfully developed the bacterial vaginosis model in rats and the hydrogel base did not show any antibacterial activity. This confirmed that the excellent efficacy in the bacterial vaginosis model was due to the combination of thermoresponsive hydrogel and gel flakes formulation.

**Figure 6:**
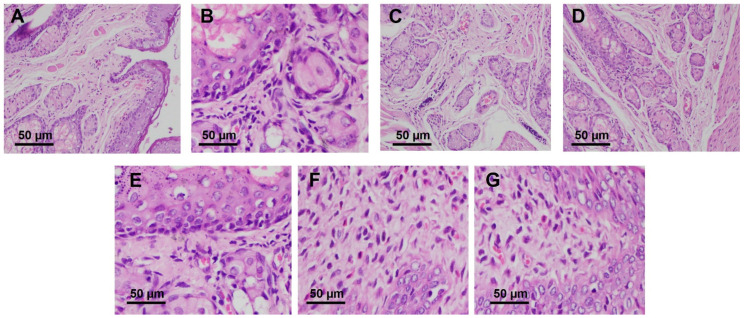
Histopathological examinations of vaginal tissue of healthy rats (**A**) in comparison with untreated rats (**B**), as well as rats receiving hydrogel containing gel flakes (**C**), hydrogel containing the free drug (**D**), hydrogel gels (**E**), free drug solution (**F**), and gel flakes dispersion (**G**).

Following the success of the antibacterial activity in the bacterial vaginosis model, it was crucial to assess the histopathological evaluation of the vaginal tissues after the administration of this approach. This could also give us the information regarding the potential irritation caused by the administration of the system developed here. As shown in Figure 6 the vaginal tissues of healthy rats were found to be free from edema, infiltration, and congestion. The same results were also found after the administration of the hydrogel containing gel flakes. On the other hand, severe irritations with edema, thinning of epithelial, infiltration, and erosion were observed in the untreated rats. The administration of the hydrogel containing free drug, free drug solution, and gel flakes dispersion was only able to heal the infection condition to moderate irritations. From these results, it was shown the combination of gel flakes and thermoresponsive hydrogel with mucoadhesion properties could not only reduce the bacterial bioburden significantly, but also heal the condition of the infected tissues to a normal condition.

## 4. Conclusions

The combinatory approach of the thermoresponsive hydrogel with mucoadhesive properties and gel flakes can offer better efficacy for the treatment of BV. Here, we successfully developed gel flakes containing metronidazole using the combination of gellan gum and chitosan. All formulations could load more than 65% of metronidazole, showing the effectiveness of encapsulation efficiency of the system developed. The formation of gel flakes was effectively observed using FTIR evaluation. Importantly, the formulation of metronidazole into gel flakes could sustain the release over 24 h. Moreover, the incorporation into a mucoadhesive-thermoresponsive hydrogel was found to be effective to treat BV in rats model, in comparison with hydrogel containing free drug, free drug solution, and gel flakes dispersion. Following the histopathological evaluation, this system could heal the infected tissue to the normal condition. This could be beneficial as an alternative for the current treatment. However, to further explore the effectiveness of this system, several studies are now needed, including stability studies and biocompatibility and toxicity evaluations prior to the next experimental steps.

## Figures and Tables

**Table 1:** Composition of gel flakes containing metronidazole.

Compounds	F1	F2	F3	F4	F5	F6
Metronidazole (%*w*/*v*)	1	1	1	1	1	1
Gellan Gum (%*w*/*v*)	0.1	0.2	0.3	0.1	0.2	0.3
Chitosan (%*w*/*v*)	0.1	0.1	0.1	0.2	0.2	0.2
Distilled water to	100	100	100	100	100	100

**Table 2:** The composition of hydrogel containing gel flakes of metronidazole (% *w*/*v*).

Formula	Gel Flakes (Equal to Pure Metronidazole)	PF-127	PF-68	Sodium Alginate
G1	1	20.00	-	-
G2	1	17.50	2.50	-
G3	1	15.00	5.00	-
G4	1	12.50	7.50	-
G5	1	10.00	10.00	-
G6	1	15.00	5.00	0.20
G7	1	15.00	5.00	0.40
G8	1	15.00	5.00	0.60

**Table 3:** The EE and DL values of metronidazole in gel flakes formulations (mean ± SD, *n* = 3).

Formulation	EE (%)	DL (%)
F1	77.67 ± 3.43	79.52 ± 2.65
F2	79.43 ± 2.12	72.59 ± 3.09
F3	84.32 ± 2.61	67.82 ± 2.42
F4	90.65 ± 2.34	75.13 ± 3.19
F5	98.91 ± 3.43	74.20 ± 3.98
F6	99.47 ± 2.98	66.55 ± 4.51

**Table 4:** The gelation temperature of thermoresponsive in situ hydrogel containing metronidazole gel flakes (mean ± S.D., *n* = 3).

	T_sol-gel_ (Without Dilution)	T_sol-gel_ (With Dilution)
G1	23.43 ± 2.32	23.91 ± 2.43
G2	29.54 ± 2.71	30.87 ± 3.01
G3	36.87 ± 3.43	37.43 ± 3.14
G4	40.54 ± 4.02	42.32 ± 4.90
G5	43.52 ± 4.11	45.41 ± 3.87
G6	36.98 ± 3.12	37.94 ± 2.87
G7	37.01 ± 3.03	37.98 ± 3.21
G8	46.76 ± 3.18	47.65 ± 4.09

**Table 5:** The mucoadhesion strength and time of thermoresponsive hydrogel (mean ± S.D., *n* = 3).

	Mucoadhesion Strength (dyne·cm^2^)	Mucoadhesion Time (h)
G1	28.32 ± 2.31	3.98 ± 0.32
G2	23.31 ± 1.98	3.18 ± 0.28
G3	18.23 ± 0.93	2.81 ± 0.43
G4	16.21 ± 1.87	2.19 ± 0.19
G5	11.23 ± 1.02	2.01 ± 0.21
G6	29.43 ± 2.32	6.53 ± 0.51
G7	42.31 ± 3.82	8.62 ± 0.72
G8	45.64 ± 4.01	8.91 ± 0.82

**Table 6:** The results of measured pH and drug content of in situ vaginal gel (mean ± S.D., *n* = 3).

	pH	Drug Content
G1	5.15 ± 0.39	98.32 ± 0.43
G2	5.21 ± 0.41	97.76 ± 0.32
G3	5.19 ± 0.54	98.43 ± 0.44
G4	5.43 ± 0.33	98.31 ± 0.63
G5	5.28 ± 0.13	99.01 ± 0.43
G6	5.34 ± 0.34	98.21 ± 0.23
G7	5.28 ± 0.27	99.34 ± 0.53
G8	5.19 ± 0.32	99.63 ± 0.65

## Data Availability

Not applicable.
